# 25(OH)D_2_ Half-Life Is Shorter Than 25(OH)D_3_ Half-Life and Is Influenced by DBP Concentration and Genotype

**DOI:** 10.1210/jc.2014-1714

**Published:** 2014-06-02

**Authors:** K. S. Jones, S. Assar, D. Harnpanich, R. Bouillon, D. Lambrechts, A. Prentice, I. Schoenmakers

**Affiliations:** Medical Research Council Human Nutrition Research (K.S.J., S.A., D.H., A.P., I.S.), Cambridge CB1 9NL, United Kingdom; Medical Research Council Keneba (K.S.J., A.P.), The Gambia; Clinic and Laboratory of Experimental Medicine and Endocrinology (R.B.) and Laboratory for Translational Genetics (D.L.), Katholieke Universiteit, B-3000 Leuven, Belgium; and Vesalius Research Center (D.L.), VIB, Katholieke Universiteit, B-3000, Leuven, Belgium

## Abstract

**Context:**

There is uncertainty over the equivalence of vitamins D_2_ and D_3_ to maintain plasma 25-hydroxyvitamin D (25(OH)D).

**Objective:**

The objective of the study was to compare the plasma half-lives of 25(OH)D_2_ and 25(OH)D_3_ in two distinct populations with different dietary calcium intake and 25(OH)D status.

**Participants:**

Healthy men (aged 24 and 39 y), resident in The Gambia (n = 18) or the United Kingdom (n = 18) participated in the study.

**Interventions:**

The intervention included an oral tracer dose of deuterated-25(OH)D_2_ and deuterated-25(OH)D_3_ (both 40 nmol). Blood samples were collected over 33 days.

**Main Outcome Measures:**

25(OH)D_2_ and 25(OH)D_3_ plasma half-lives, concentrations of 25(OH)D, and vitamin D binding protein (DBP) and *DBP* genotypes were measured.

**Results:**

25(OH)D_2_ half-life [mean (SD)] [13.9 (2.6) d] was shorter than 25(OH)D_3_ half-life [15.1 (3.1) d; *P* = .001] for countries combined, and in Gambians [12.8 (2.3) d vs 14.7 (3.5) d; *P* < .001], but not in the United Kingdom [15.1 (2.4) d vs 15.6 (2.5) d; *P* = .3]. 25(OH)D concentration was 69 (13) and 29 (11) nmol/L (*P* < .0001), and the DBP concentration was 259 (33) and 269 (23) mg/L (*P* = .4) in The Gambia and United Kingdom, respectively. Half-lives were positively associated with plasma DBP concentration for countries combined [25(OH)D_2_ half-life: regression coefficient (SE) 0.03 (0.01) d per 1 mg/L DBP, *P* = .03; 25(OH)D_3_ half-life: 0.04 (0.02) d, *P* = .02] and in Gambians [25(OH)D_2_ half-life: 0.04 (0.01) d; *P* = .02; 25(OH)D_3_ half-life: 0.06 (0.02) d, *P* = .01] but not in UK participants. The DBP concentration × country interactions were not significant. DBP Gc1f/1f homozygotes had shorter 25(OH)D_2_ half-lives compared with other combined genotypes (*P* = .007) after correction for country.

**Conclusions:**

25(OH)D_2_ half-life was shorter than 25(OH)D_3_ half-life, and half-lives were affected by DBP concentration and genotype. The stable isotope 25(OH)D half-life measurements provide a novel tool to investigate vitamin D metabolism and vitamin D expenditure and aid in the assessment of vitamin D requirements.

Vitamin D is essential for human health, and certain groups may require supplementation to prevent vitamin D deficiency (1). Vitamin D_3_ (cholecalciferol) is formed endogenously in the skin on exposure to UVB light and is also available from some foods, either naturally or through fortification. Vitamin D_2_ (ergocalciferol) is present in some fortified foods, supplements, and a small number of natural foods. Either form of the vitamin is used for prophylaxis and/or treatment. However, there is uncertainty over the relative effectiveness of the two forms of vitamin D (2, 3).

Both vitamin D_2_ and vitamin D_3_ are effective in the prevention and treatment of vitamin D deficiency rickets and osteomalacia (4–6). Some studies have noted differences in the PTH response after single oral doses of vitamin D_2_ or vitamin D_3_ (7, 8), whereas others, with regular oral doses, have reported no difference in changes in PTH levels (4, 9, 10), bone turnover markers (4), or calcium absorption (11). Less clear is the ability of vitamin D_2_ compared with vitamin D_3_ to maintain plasma 25-hydroxyvitamin D [25(OH)D], particularly after a single bolus dose. The initial rise in plasma 25(OH)D concentration in response to a single dose of vitamin D_2_ or vitamin D_3_ is similar (7), but the subsequent decline may be more rapid after a vitamin D_2_ dose (2, 3, 7). Differences in the 25(OH)D plasma response to vitamin D_2_ and vitamin D_3_ may be due to differences between vitamin D_2_ and vitamin D_3_ or their metabolites in affinity for vitamin D binding protein (DBP), hydroxylases, or the vitamin D receptor (VDR). Vitamin D metabolites are primarily transported in plasma by DBP, and their binding affinities for DBP, which may also be altered by genetic variation in DBP, are important determinants of plasma half-life (12). Accordingly, half-lives of vitamin D and 1,25-dihydroxyvitamin D [1,25(OH)_2_D] are shorter than that of 25(OH)D. Similarly, a shorter half-life for vitamin D_2_ metabolites may be expected due to their lower DBP binding affinities (13).

We have developed a method to measure 25(OH)D plasma half-life using stable isotope-labeled compounds. The purpose of this experimental study was to measure the plasma half-lives of 25(OH)D_2_ and 25(OH)D_3_ simultaneously. The study was performed in a rural West African setting and in Cambridge, United Kingdom, countries that differ markedly in vitamin D status, calcium intake, and markers of vitamin D metabolism [eg, 1,25(OH)_2_D and PTH] (14). In addition, these groups differ in their predominant *DBP* genotypes (15). Therefore, performing the study in these two populations provided contrasting environments to investigate environmental and genetic influences on vitamin D metabolism.

## Materials and Methods

### Study setting

The study took place in May and June 2010 at the rural field station of the UK Medical Research Council (MRC) in Keneba, The Gambia (latitude 13°N), where there is little seasonal variability in vitamin D synthesis or status (16), and at MRC Human Nutrition Research, Cambridge, United Kingdom (latitude 52°N) between January and April 2011, when there is little cutaneous vitamin D synthesis. The study was conducted according to the Declaration of Helsinki. In each country, trained staff explained the study to participants and informed, written consent was obtained. All procedures were approved by the joint Gambian Government-MRC Ethics Committee or the UK National Research Ethics Service, Cambridge Committee.

### Participants

Thirty-six healthy, nonsmoking males, aged 24–39 years, with a body mass index (BMI) less than 27 kg/m^2^ participated. One additional Gambian participant was removed from the analysis because his estimated half-lives were more than 3 interquartile ranges beyond the 75th centile and had a major impact on the relationships between the half-life and other variables. Their 25(OH)D, 1,25(OH)_2_D, and 24,25-dihydroxyvitamin D [24,25(OH)_2_D] plasma concentrations were typical of the other participants and suggested nothing abnormal about their vitamin D metabolism. Plasma tracer concentrations were, however, variable across the time course of the experiment, suggesting the long half-life was an artifact.

Exclusion criteria were recent illness (within 2 weeks); broken bone within the last 3 years; known bone, kidney, or liver disease; taking any prescription medicines; or a hemoglobin level less than 10 g/dL. UK participants were all self-classified as white European and black Gambians were all of Mandinka ethnicity.

### Dose preparation

Deuterated (three deuterium atoms at positions 6, 19, and 19) 25(OH)D_2_ [d_3_-25(OH)D_2_] and 25(OH)D_3_ [d_3_-25(OH)D_3_] (product numbers 705497 and 705888) (both 97 atom percentage; purity 98%; Sigma-Aldrich) were dissolved in vegetable oil. The solution was protected from light and incubated in a water bath for 1 hour at 35°C and then mixed thoroughly. Aliquots were frozen at −20°C until use. Each 1000 μL dose contained 40 nmol of both deuterated 25(OH)D_2_ and deuterated 25(OH)D_3_.

### Study protocols

The same study protocols were followed in The Gambia and the United Kingdom. On day 1 and day 21 of the study and after an overnight fast and voiding of the first morning urine, a 2-hour fasting urine was collected from approximately 7:00 am as described previously (17). EDTA and lithium-heparin (LH) (Sarstedt Ltd) blood samples were collected after 1 hour and placed on ice. Height (Leicester Stadiometer; Chasmoors Ltd) and weight (Tanita HD305 scale; Chasmoors Ltd) were measured. After completion of the urine collection, the dose was pipetted onto a small piece of bread and eaten by the participant under supervision, followed by a standardized breakfast (17). Breakfasts were different between countries but were designed to have equal energy content and percentage energy from fat, protein, and carbohydrate. Water was permitted ad libitum. After the dose, participants were asked to refrain from lying down, exercising, or eating for the following 5 hours. Fasted blood samples were collected on day 6 and (±2 d) days 9, 21, 24, 27, 30, and 33 to measure plasma half-lives (17).

### Sample processing and laboratory analyses

Plasma and urine were treated and stored as described previously (17). Samples from The Gambia were shipped on dry ice to MRC Human Nutrition Research for analysis. EDTA plasma was used for analysis of PTH in singleton by immunoassay (Immulite; Siemens Healthcare Diagnostics Ltd). Between-assay coefficient of variation (CV) was 4.7%. All other assays were performed in duplicate with LH plasma. Albumin was measured on the Konelab 20i (Kone). Within- and between-assay CVs were less than 2% and less than 4%, respectively. Within- and between-assay CVs for total plasma 1,25(OH)_2_D were 7.5% and 9.0% (IDS Ltd). Performance was monitored using kit and in-house controls and under strict standardization according to ISO 9001:2000. Quality assurance of 25(OH)D, 1,25(OH)_2_D, and PTH assays were performed as part of the Vitamin D External Quality Assessment Scheme (www.deqas.org) and the National External Quality Assessment Scheme (www.ukneqas.org.uk) and were within acceptable limits. DBP was measured by a radial immunodiffusion assay with a polyclonal antibody (18), and 24,25(OH)_2_D was analyzed by ultraperformance liquid chromatography and tandem mass spectrometry (UPLC-MS/MS) (19) at Katholieke Universiteit, Leuven, Belgium.

Derivatized 25(OH)D_2_/D_3_ and tracers were measured by UPLC-MS/MS as described previously (17, 20). Tracer and endogenous 25(OH)D_2_/D_3_ extraction and analysis were performed separately with different levels of internal standards, d_6_-25(OH)D_2_ and d_6_-25(OH)D_3_ (Chemaphor Inc). 25(OH)D_2_, 25(OH)D_3_, d_3_-25(OH)D_2_, and d_3_-25(OH)D_3_ were purchased from Sigma-Aldrich. Plasma [200 μL for 25(OH)D_3_/D_2_ and 250 μL for tracers] was mixed with 500 or 600 μL acetonitrile, respectively, to release protein-bound vitamin D metabolites. Sample pretreatment and UPLC-MS/MS operating parameters were as described previously (20) with slight modifications applied for the Acquity ultraperformance liquid chromatography module (Waters), interfaced to a 3200 tandem mass spectrometer (AB Sciex). Mass transitions were (mass to charge ratio) 607→298 for 25(OH)D_3_, 619→298 for 25(OH)D_2_, 610→301 for d_3_-25(OH)D_3_, 622→301 for d_3_-25(OH)D_2_, 613→298 for d_6_-25(OH)D_3_, and 625→298 for d_6_-25(OH)D_2_. Calibrations used a seven-point standard curve containing 25(OH)D_3_ (0–130 nM), 25(OH)D_2_ (0–12 nM), or tracers (0–4 nM). Intra- and interassay CVs were less than 10%.

### Genetic analysis

DNA was extracted from LH blood pellets with QIAGEN QIAamp DNA Blood maxi kit. The genotyping of *DBP* and vitamin D-hydroxylase gene variants known to be associated with vitamin D status and of well-described *VDR* variants was performed at the Vesalius Research Center (Katholieke Universiteit, Leuven, Belgium) by iPLEX technology on a MassARRAY compact analyzer (Sequenom Inc) (21).

### Derived variables and data analysis

The slope of plasma tracer disappearance (k_B_) was calculated from the line of best fit of the natural log of d_3_-25(OH)D_2_ and d_3_-25(OH)D_3_ concentrations against time from day 6 to day 33. Half-lives were then calculated in Microsoft Excel 2010 (Microsoft Corp) using the following equation:

$$T\frac{1}{2}=\frac{\text{In}\left(2\right)}{k_{B}}$$

Statistics were performed in Datadesk 6.3 (Data Description, Inc). Normally distributed data are presented as mean and SD. Skewed data were log transformed and are presented as geometric means and 95% confidence interval. Total 25(OH)D concentration [t25(OH)D] was the sum of 25(OH)D_2_ and 25(OH)D_3_.

Country differences in half-lives were examined using a linear model with ln d_3_-25(OH)D concentration as the dependent variable and participant, country and sample time point as independent variables, with an interaction term between country and participant. In paired Student's *t* tests, there were no differences between day 1 and day 21 values, so biochemical data were used in statistical analysis and are presented as the day 1–21 mean. DBP and 24,25(OH)_2_D were measured on day 21 and day 1 only, respectively. Country differences were determined using unpaired *t* tests. Associations between half-lives and t25(OH)D, DBP, bioavailable 25(OH)D [b25(OH)D], or free-25(OH)D [f25(OH)D] were explored using linear regression and with interaction terms between the independent variable and country [eg, t25(OH)D × country]. Results were considered significant when *P* < .05.

f25(OH)D (nonprotein bound) and b25(OH)D (free and albumin-bound portion) were calculated according to Powe et al (22) and Chun et al (23). The model by Chun et al also accounts for affinity differences due to *DBP* genotype [Gc-f25(OH)D and Gc-b25(OH)D], but both models use the same association constants for DBP and albumin. DBP isoforms (haplotypes) were defined on the basis of allele combinations of rs7041 and rs4588 single nucleotide polymorphisms (SNPs): Gc1f, T-C; Gc1s, G-C, and Gc2, T-A. To investigate the relationships between half-lives, DBP concentration, and t25OHD with relevant SNPs (21), we used ANOVA with Scheffé post hoc tests and analysis of covariance with the inclusion of country as a confounder (see Table 3). Due to the small group sizes, the relationships with half-life were also examined by combined genotype (diplotype), ie, Gc1f/1f (T-C/T-C) diplotype vs the other diplotypes combined [(Gc1f/1s (T-C, G-C) (n = 10), Gc1f/2 (T-C, T-A) (n = 2), Gc1s/1s (G-C, G-C) (n = 6), and Gc1s/2 (G-C, T-A) (n = 1)].

## Results

**Figure 1.**
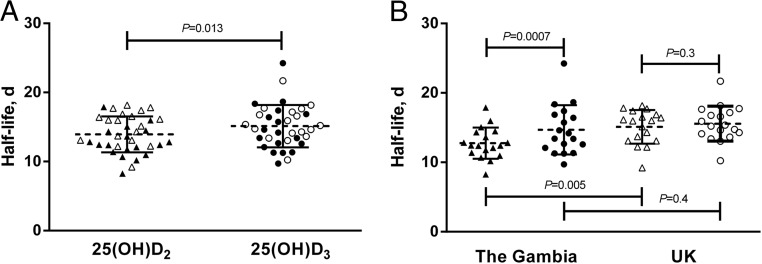
Mean, SD, and individual values for the half-lives of 25(OH)D_2_ (▴) and 25(OH)D_3_ (●) for countries combined [18 Gambian (solid symbols) and 18 UK (open symbols) participants] (A) and the countries separated (B). Differences between the half-lives of 25(OH)D_2_ and 25(OH)D_3_ and country differences are displayed as *P* values.

Differences between countries were observed in most biochemical parameters including t25(OH)D, 1,25(OH)_2_D, and PTH (Table 1). There was no difference in the DBP concentration between countries, but f25(OH)D and b25(OH)D were significantly higher in Gambian participants and albumin significantly lower (Table 1). Country differences in tracer concentration at the same time points revealed significant differences at day 30 for 25(OH)D_2_ (*P* = .01) and day 33 for 25(OH)D_3_ (*P* = .02). The 25(OH)D_2_ half-life was significantly shorter than the 25(OH)D_3_ half-life for the countries combined, 13.9 (2.6) days and 15.1 (3.1) days, respectively (*P* = .001) (Figure 1A). When countries were analyzed separately, there was a significant difference between 25(OH)D_2_ and 25(OH)D_3_ half-lives in Gambian (*P* = .0007) but not UK participants (*P* = .3) (Figure 1B).

**Table 1.** Anthropometric and Biochemical Characteristics for 18 Gambian and 18 UK Participants and Country Differences^a^

	The Gambia	UK	*P* Value^b^
Age, y	29.2 (3.2)	29.3 (4.4)	1.00
Weight, kg	64.5 (8.3)	73.3 (10.9)	.01
Height, m	1.74 (0.06)	1.80 (0.07)	<.01
BMI, kg/m^2^	21.3 (2.0)	22.6 (2.3)	.08
25(OH)D_2_, nmol/L	0.6 (0.2)	2.1 (1.0)	<.0001
25(OH)D_3_, nmol/L	68.4 (13.1)	26.5 (10.8)	<.0001
t25(OH)D, nmol/L	69.0 (13.2)	28.6 (11.0)	<.0001
1,25(OH)_2_D, pmol/L^c^	181 (165, 197)	120 (109, 132)	<.0001
24,25(OH)_2_D, nmol/L^d^	6.5 (1.3)	1.9 (1.4)	<.0001
PTH, μg/L^c^	50.1 (41.9, 60.0)	32.8 (27.4, 39.3)	<.01
DBP, mg/L^d^	259 (33)	268 (23)	.4
Albumin, g/L	36.4 (2.2)	41.0 (2.4)	<.0001
b25(OH)D, nmol/L	6.7 (1.2)^e^	3.0 (1.3)^f^	<.0001
f25(OH)D, pmol/L	20.4 (4.2)^e^	8.1 (3.3)^f^	<.0001
Gc-b25(OH)D, nmol/L^g^	6.6 (1.6)^e^	4.4 (2.1)^f^	.005
Gc-f25(OH)D, pmol/L^g^	20.1 (5.5)^e^	12.0 (5.6)^f^	.0008

Abbreviations: Gc-b25(OH)D, Gc-genotype-corrected bioavailable 25(OH)D; Gc-f25(OH)D, Gc-genotype-corrected free 25(OH)D.

a Other than age, weight, height, and BMI, data are presented as means (SD) of data collected on days 1 and 21 unless marked.

b Country differences as tested by unpaired, two-tailed Student's *t* test.

c Geometric means and 95% confidence interval are given.

d 24,25(OH)_2_D and DBP were measured at one time point only.

e There were no differences between f25(OH)D and Gc-f25(OH)D or b25(OH)D and Gc-b25(OH)D in Gambians (both *P* = .9).

f In UK participants, Gc-f25(OH)D and Gc-b25(OH)D were significantly higher than f25(OH)D and b25(OH)D (*P* = .003 and *P* = .002, respectively).

g Calculated on a subset of the population (Gambia, n = 16; UK, n = 12).

**Figure 2.**
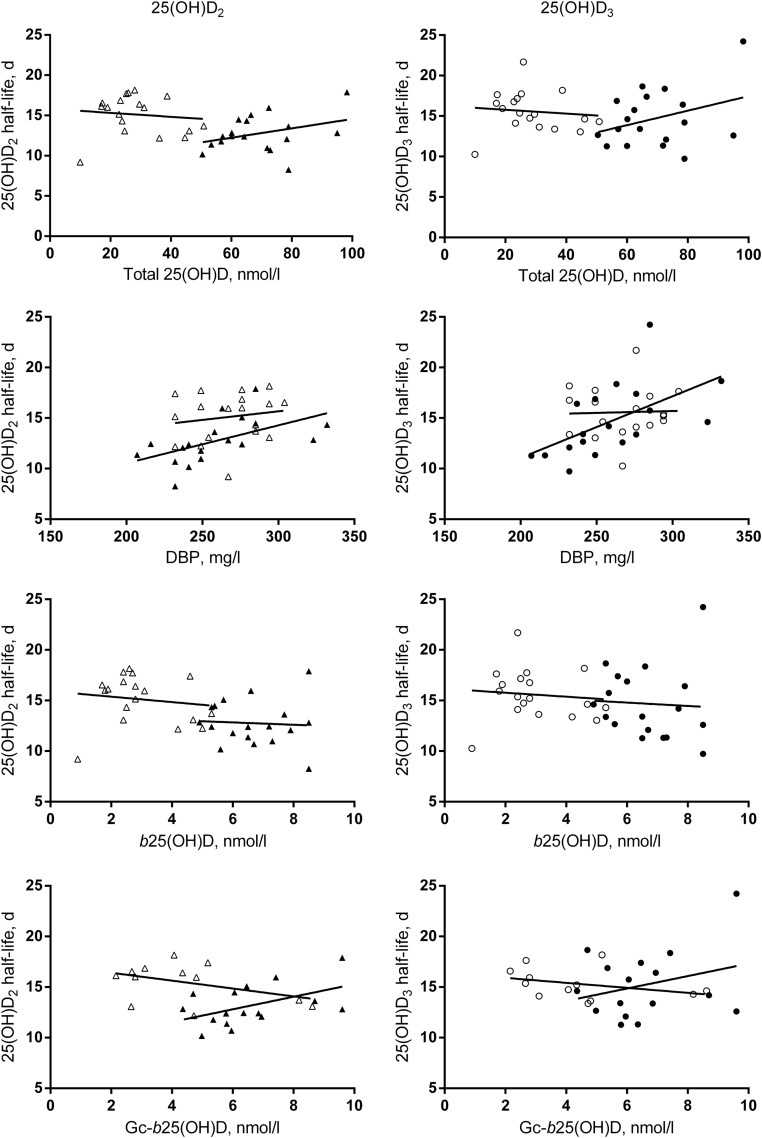
Country-specific relationships between the half-lives and total 25(OH)D, DBP, and b25(OH)D concentrations for 25(OH)D_2_ (▴, Gambia; ▵, UK) and 25(OH)D_3_ (●, Gambia; ○, UK) in 18 Gambian and 18 UK participants, and Gc-b25(OH)D in 16 Gambian and 12 UK participants. The charts show individual values and country-specific regression lines. Regression line statistics are shown in Table 2.

Comparison of half-lives between countries revealed that the 25(OH)D_2_ half-life was significantly shorter [mean difference (SE)] [2.3 (0.8) day ; *P* = .005] in Gambian participants than in UK participants, but there was no difference in the 25(OH)D_3_ half-life [0.9 (1.0) d; *P* = .4) (Figure 1B]. In the regression analysis, there were no significant relationships between half-lives and t25(OH)D, f25(OH)D, or b25(OH)D (Table 2). Relationships between half-lives and the f25(OH)D index [the molar ratio of t25(OH)D and DBP concentrations (24)] (data not shown) were very similar to those reported for f25(OH)D and b25(OH)D. Plasma DBP concentration was significantly and positively associated with both 25(OH)D_2_ and 25(OH)D_3_ half-lives in Gambians and for the countries combined but not in the UK participants (Figure 2 and Table 2). However, DBP concentration × country interactions were not significant. DBP isoform (haplotype) frequencies differed between countries (χ^2^ test, *P* < .0001) [Gc1f (T-C): 0.78 vs 0.21; Gc1s (G-C): 0.19 vs 0.71; Gc2 (T-A): 0.03 vs 0.08 for The Gambia and the United Kingdom, respectively].

**Table 2.** Linear Regression Data for Associations Between Half-Lives and t25(OH)D, DBP, and b25(OH)D and f25(OH)D in 18 Gambian and 18 UK Participants

	25(OH)D_2_ Half-Life, d		25(OH)D_3_ Half-Life, d	
	β-Coefficient (SE)	*P* Value	β-Coefficient (SE)	*P* Value
t25(OH)D, nmol/L·U				
Gambia	0.058 (0.039)	.2	0.090 (0.063)	.2
United Kingdom	−0.024 (0.055)	.7	−0.024 (0.057)	.7
Countries combined	0.025 (0.033)	.5	−0.043 (0.043)	.3
Country		.04		.2
25(OH)D*country interaction		.2		.2
DBP, mg/L				
Gambia	0.037 (0.014)	.02	0.061 (0.022)	.01
United Kingdom	0.017 (0.026)	.5	0.004 (0.027)	.9
Countries combined	0.031 (0.013)	.03	0.042 (0.017)	.02
Country		.008		.6
DBP*country interaction		.1		.1
b25(OH)D, nmol/L				
Gambia	−0.116 (0.469)	.8	−0.166 (0.740)	.8
United Kingdom	−0.268 (0.481)	.6	−0.198 (0.500)	.7
Countries combined	−0.192 (0.337)	.6	−0.182 (0.436)	.7
Country		.3		.9
b25(OH)D*country interaction		.8		1.00
f25(OH)D, pmol/L				
Gambia	−452e-6 (0.134)	1.00	−0.010 (0.211)	1.00
United Kingdom	0.100 (0.181)	.6	−0.075 (0.187)	.7
Countries combined	−0.039 (0.107)	.7	−0.035 (0.141)	.8
Country		.2		.8
f25(OH)D*country interaction		.7		.8
Gc-b25(OH)D, nmol/L				
Gambia	0.621 (0.292)	.052	0.615 (0.547)	.3
United Kingdom	−0.389 (0.173)	.2	−0.247 (0.215)	.3
Countries combined	0.056 (0.216)	.8	0.133 (0.303)	.7
Country		.01		.8
Gc-b25(OH)D*country interaction		.02		.2
Gc-f25(OH)D, pmol/L				
Gambia	0.175 (0.086)	.06	0.173 (0.160)	.3
United Kingdom	−0.143 (0.094)	.2	−0.092 (0.077)	.3
Countries combined	0.030 (0.070)	.7	0.052 (0.098)	.6
Country		.01		.7
Gc-f25(OH)D*country interaction		.02		.2

Abbreviations: Gc-b25(OH)D, Gc-genotype-corrected bioavailable 25(OH)D; Gc-f25(OH)D, Gc-genotype -corrected free 25(OH)D.

25(OH)D_2_ half-lives were significantly different between the rs7041 genotypes and the combined genotypes (Table 3), and there was a trend for the same pattern for 25(OH)D_3_ half-life; inclusion of country decreased the significance of the relationships with 25(OH)D_2_. t25(OH)D was significantly different between genotypes, and this was strengthened by correction for country (Table 3). Gc-f25(OH)D and Gc-b25(OH)D were similar to f25(OH)D and b25(OH)D in the Gambians but were higher in the UK participants (Table 1). Relationships between half-lives and either f25(OH)D and b25(OH)D or Gc-f25(OH)D and Gc-b25(OH)D were similar (Figure 2 and Table 2), but relationships were different between countries for the 25(OH)D_2_ half-life and Gc-f25(OH)D and Gc-b25(OH)D as confirmed by the significant country interaction (Table 2).

**Table 3.** Genotype Frequencies and 25(OH)D_2_ and 25(OH)D_3_ Half-Lives, t25(OH)D and DBP Concentration Group Values and Differences by Genotype^a^

Gene, Variant	Genotype	n^b^	25(OH)D_2_ Half-Life, d	*P* Value^a^	25(OH)D_3_ Half-Life, d	*P* Value^a^	t25(OH)D, nmol/L	*P* Value^a^	DBP mg/L	*P* Value^a^
*DBP*, rs7041	TT	11	12.5 (1.8)	.0009 (.052)^c^	14.1 (2.4)	.07 (.07)	58.3 (14.2)	.03 (.02)^d^	257 (41)	.1 (.2)
	GT	13	14.9 (2.0)		16.6 (2.9)		52.7 (28.0)		264 (20)	
	GG	6	16.1 (1.4)		15.2 (1.4)		29.3 (12.0)		287 (14)	
	Country			.3		.6		<.0001		.6
*DBP*, rs4588	CC	27	13.7 (2.6)	.8 (.9)	15.0 (3.1)	.6 (.6)	52.4 (24.6)	.5 (.9)	264 (32)	.8 (.9)
	CA	3	14.1 (2.4)		14.0 (0.6)		42.2 (17.2)		269 (13)	
	AA	0								
	Country			.03		.9		<.0001		.3
*DBP*, Gc1f/1f (TT, CC)	1f/1f^e^	9	12.0 (1.2)	.0001 (.007)	14.1 (2.7)	.1 (.08)	62.4 (9.0)	.1 (.03)	253 (44)	.1 (.2)
	Others^e^	19	15.1 (1.9)		15.8 (2.7)		47.0 (25.8)		272 (20)	
	Country			.3		.3		<.0001		.9
*CYP24A1*, rs6013897	TT	17	14.8 (2.3)	.2 (.08)	15.9 (3.1)	.1 (.1)	55.3 (26.2)	.4 (.2)		
	AT	11	13.5 (2.9)		14.1 (2.4)		47.7 (19.9)			
	AA	0								
	Country			.0003		.3		<.0001		
*CYP2R1*, rs10741657	GG	10	13.7 (2.9)	.3 (.4)	15.9 (3.8)	.6 (.5)	64.4 (18.5)	.02 (.95)^f^		
	GA	16	13.7 (2.1)		14.8 (2.4)		43.3 (22.6)			
	AA	2	16.1 (0.6)		13.9 (0.3)		27.2 (5.5)			
	Country			.08		.7		<.0001		
*VDR*, rs731236 (Taq1)	TT	11	12.8 (2.1)	.1 (.5)	14.3 (2.9)	.4 (.5)	59.6 (19.4)	.2 (.6)		
	TC	18	14.6 (2.8)		15.5 (3.1)		47.6 (25.7)			
	CC	1	16.5 (0)		17.6 (0)		17.3 (0)			
	Country			.03		.9		<.0001		
*VDR*, rs739837 (Apa1)	TT	8	14.0 (2.3)	.6 (.8)	14.6 (2.3)	.4 (.4)	52.7 (21.7)	1.00 (.4)		
	GT	23	14.0 (2.7)		15.4 (3.1)		48.9 (25.8)			
	GG	1	11.4 (0)		11.3 (0)		53.3 (0)			
	Country			.02		.9		<.0001		
*VDR*, rs10735810 (Fok1)	CC	16	13.4 (2.5)	.3 (.5)	14.1 (2.4)	.06 (.09)	59.6 (21.1)	.08 (.3)		
	TC	13	15.0 (2.5)		16.6 (2.9)		40.7 (23.7)			
	TT	1	14.5 (0)		15.5 (0)		62.4 (0)			
	Country			.002		.8				

a ANOVA for genotype differences by genotype and Scheffé post hoc test for paired differences. Bracketed *P* values are for analysis of covariance models that include country.

b DNA was available for 35 participants, but low DNA yield prevented genotyping in three individuals. The remaining variation in sample sizes is due to not all SNPs being called.

c TT vs GT, *P* = .01; TT vs GG, *P* = .002.

d TT vs GG, *P* = .04.

e Gc1f/1f combined genotype is assigned by TT and CC in SNPs rs7041 and rs4588, respectively. “Others” consists of Gc1f/1s (TG, CC) (n = 10), Gc1f/2 (TT, CA) (n = 2), Gc1s/1s (GG, CC) (n = 6), and Gc1s/2 (TG, CA) (n = 1).

f No significant individual comparisons (GG vs GA, *P* = 0.06; GG vs AA, *P* = 0.09).

## Discussion

25(OH)D half-life is a measure of 25(OH)D expenditure and is determined by CYP27B1 and CYP24A1 enzyme activity and factors that affect 25(OH)D transport and delivery to cells. In this study 25(OH)D_2_ half-life was shorter than 25(OH)D_3_ half-life for the countries combined and in Gambian participants when the countries were examined separately. 25(OH)D_2_ half-life, but not 25(OH)D_3_ half-life, was shorter in the Gambian compared with the UK participants. The DBP concentration significantly predicted 25(OH)D_2_ and 25(OH)D_3_ half-lives in the combined and Gambian models but not in the UK participants, although the DBP concentration country interaction was not significant. These different country relationships may be related to differences in DBP genotype.

These data may partly explain the previous findings that equal doses of vitamin D_2_ and vitamin D_3_ do not equally maintain plasma 25(OH)D. The affinity of vitamin D_2_ metabolites for DBP is lower than that of vitamin D_3_ metabolites (13) and may result in proportionally higher f25(OH)D_2_ available for metabolism. This, together with differences in hydroxylase affinity for vitamin D_2_ and vitamin D_3_ metabolites, may explain the shorter half-life of 25(OH)D_2_ compared with 25(OH)D_3_. However, our data also suggest that these differences may not be consistent between populations, whether due to environmental or genetic factors. Our data support the assertion that the initial 25(OH)D plasma response after doses of vitamin D_2_ and vitamin D_3_ is comparable (7), reflecting similar intestinal absorption, but that differences in attained 25(OH)D concentration are observed after a period of days or weeks (3, 4, 7, 8, 25), suggesting differences in clearance. However, these observations may also be related to dosing frequency because studies that have given daily doses of vitamin D_2_ or vitamin D_3_ have observed a small (4) or no (2, 10, 26) difference in the 25(OH)D response.

Other factors may also influence the conclusions of these earlier studies and include inadequate analytical specificity, not controlling for UVB exposure and the use of large doses (26), as well as different dose vehicles (4, 7, 25) or foods (10, 26). Labeled tracer compounds can be differentiated from endogenous vitamin D and permit the use of small doses that do not perturb normal calcium, phosphate, or vitamin D metabolism or status (17). The nature of intervention studies and the difficulty in performing crossover studies in which there is a strong seasonal influence has meant that earlier studies have not directly compared vitamin D_2_ and vitamin D_3_ or 25(OH)D_2_ and 25(OH)D_3_. In this study, tracers were administered together and thereby interindividual and seasonal effects were eliminated.

DBP concentration and genotype may modify vitamin D metabolism and function, eg, affecting bone mineral density (22) and immune cell activity (23). Two SNPs in the *DBP* gene, rs4588 and rs7041, give rise to three polymorphic isoforms, designated Gc1s, Gc1f, and Gc2, that may predict plasma DBP and 25(OH)D concentrations (27). The biological relevance of differences in binding affinity between these isoforms is not clear (23, 28). We found differences in half-lives, t25(OH)D and DBP concentration when analyzed by *DBP* genotype. Gc1f/1f homozygotes, hypothetically considered to have the highest binding affinity for 25(OH)D, had a shorter half-life. Associations with genetic variability in *DBP* may be confounded by race (29). In our study, although all Gc1f/1f homozygotes were Gambian, the difference between genotypes remained significant for 25(OH)D_2_ half-life after correction for t25OHD, DBP concentration, and country. *DBP* genotypes are hypothesized to vary between populations as an adaptation to lower UVB exposures at higher latitudes (15). In our study, DBP isoform frequencies were similar to those published previously from the same countries (15) with a higher frequency of Gc1f in Gambians and a higher frequency of Gc1s in the UK participants. The Gambian participants had a higher 25(OH)D status, and the UK group, with the supposedly lower-affinity genotype and expected lower UVB exposure, a lower 25(OH)D status. The different relationships between half-life and DBP concentration between these ethnic groups may be related to differences in the frequencies of the *DBP* genotypes and in the vitamin D supply and/or modified by the relationship between them (29, 30).

Free-25(OH)D or b25(OH)D have been suggested as better markers of vitamin D function than t25(OH)D (31) and are calculated using DBP and albumin concentrations and affinity constants for 25(OH)D. Correction factors may also be included for *DBP* genotype (23). Gambian participants had higher calculated f25(OH)D and b25(OH)D because of higher plasma t25(OH)D and lower plasma albumin, but as a proportion of t25(OH)D, there was no difference between the countries, and this may explain the absence of larger differences in the 25(OH)D_3_ half-life between countries. In contrast to other reports (32), DBP concentration did not influence country differences in f25(OH)D and b25(OH)D. Inclusion of *DBP* genotype increased estimates of f25(OH)D and b25(OH)D in the UK group, which had a lower frequency of the Gc1f allele but had little effect on the levels in The Gambia. However, differences between countries remained. The associations between half-lives and DBP concentration and *DBP* genotype, rather than t25(OH)D, f25(OH)D, or b25(OH)D, suggest that DBP concentration and genotype are more important determinants of the 25(OH)D half-life than t25(OH)D. When corrected for genotype, there was a trend for a positive association between the Gc-f25(OH)D, Gc-b25(OH)D, and 25(OH)D_2_ half-life in the Gambian participants. The lack of a relationship with f25(OH)D, and a relationship with DBP concentration, may be explained by an overall higher 25(OH)D expenditure through pathways that are dependent on internalization of the 25(OH)D-DBP complex, such as in the kidney and muscle, rather than pathways dependent on f25(OH)D (eg, immune cells).

We hypothesized that the lower calcium intake, higher plasma PTH, 1,25(OH)_2_D, and 25(OH)D in the Gambian population (14) would result in higher production rates of both 1,25(OH)_2_D and 24,25(OH)_2_D and consequently a shorter 25(OH)D half-life (33–35). Although 1,25(OH)_2_D and 24,25(OH)_2_D were higher in Gambians, we observed a shorter half-life for 25(OH)D_2_ only. This may be because differences were detected more readily with 25(OH)D_2_ due to its lower binding affinity for DBP, which may be more affected by genetic variation in *DBP* or to differences in the rates of hydroxylation. It might be hypothesized that in The Gambia, the more common, higher-affinity Gc1f-variant may influence the availability of vitamin D and 25(OH)D for hepatic hydroxylation to 25(OH)D and renal production of 1,25(OH)_2_D, respectively, to ensure 25(OH)D supply in an environment with low calcium intake.

Half-lives measured in this, and our previous study (17) (10–24 days), tended toward the lower end of those previously reported in healthy individuals using radiolabeled tracers (summarized in reference 17). These differences may be due to differences in metabolism, dose used, timing of sample collection, or analytical methods. They are also shorter than estimates of tissue 25(OH)D half-life of approximately 3 months from depletion-type experiments in male submariners (36). However, a longer half-life might be expected in depletion studies because of the reduction in 24,25(OH)_2_D production when 25(OH)D concentration decreases (14, 37) and because it is likely that 25(OH)D produced or released from body stores (38) may contribute to the plasma pool, thereby lengthening the estimated half-life. Differences between tracer and depletion experiments may also be related to the extent of tracer equilibration between body pools (39). Our working model for half-life estimations using tracers consists of two exchanging pools that could be identified as the plasma pool and extravascular pool, between which there is assumed to be free exchange of protein-bound 25(OH)D. In contrast, studies performed over a longer period may include the mobilization of 25(OH)D from a third, deeper body pool and/or synthesis of 25(OH)D from stored vitamin D (38, 40). However, such differences in methodology should not affect the utility of shorter-term tracer methods to investigate changes in 25(OH)D half-life due to, for example, acute or chronic changes in 1,25(OH)_2_D production (34).

This study has limitations. The sample size was small and may have limited the ability to find significant relationships, particularly for different genotypes. Based on our previous study with 25(OH)D_2_ (17), this study was powered to detect a difference of 2.5 or more days between countries (5% significance and 80% power). To reduce variance due to factors other than those directly related to vitamin D metabolism, we restricted our study to healthy, young men of similar BMI. Analyses may be confounded by the associations between country, genotype, and 25(OH)D status, among others. We have not compared calculated f25(OH)D with direct measurements, but confirmation of differences in f25(OH)D_2_ and f25(OH)D_3_ would be difficult. Further studies are required to determine whether vitamin D metabolism is affected by sex, age, diet, or body composition, and larger studies are necessary to confirm the influence of genetic polymorphisms suggested in this study.

In conclusion, our results suggest there are differences in 25(OH)D_2_ and 25(OH)D_3_ plasma half-life, but these differences may differ between populations. DBP concentration and genotype may influence the 25(OH)D half-life. It is likely that both DBP-mediated (including renal/endocrine effects) and f25(OH)D cellular uptake (extrarenal) are reflected in measures of vitamin D expenditure. The dual-isotope approach allowed the direct comparison of 25(OH)D_2_ and 25(OH)D_3_ half-lives in the same individual. Oral doses, relatively infrequent sampling, and small sample volumes make this a field-friendly method that could be applied to larger cohorts. These factors, combined with sensitive and specific liquid chromatography and tandem mass spectrometry, provide a robust method with which to explore vitamin D metabolism.
